# Adaptation of Coronavirus Disease (COVID-19) Protocols to a Parisian Maternity Unit During the 2020 Pandemic: A Managerial Perspective

**DOI:** 10.1017/dmp.2020.234

**Published:** 2020-07-14

**Authors:** Ali Ghanchi

**Affiliations:** Université de Paris, CRESS, INSERM, INRA, Paris; and Service d’Obstétrique, Maternité, chirurgie médecine et imagerie fœtales, APHP, Hôpital Necker Enfants Malades, Paris

**Keywords:** COVID-19 pandemic, infection prevention and control policies, midwifery unit management, Paris, traffic control bundling

## Abstract

The coronavirus disease (COVID-19) pandemic overwhelmed health services in France during March 2020 and, to cope, service delivery was reduced in most disciplines. However, as this was impossible for Obstetrics, the COVID-19 infection had to be added to existing clinical care pathways at the children’s hospital, Hôpital Necker–Enfants Malades. This was further complicated by an increasing number of pregnancies affected by infection, in addition to scientific uncertainty about the virus. Procedures based on scientific recommendations from French and international authorities were adapted to maternity care and regularly updated as the situation progressed. Weekly medical manager team meetings covered the evolving clinical situation, and an initial evaluation revealed that our procedures worked well. However, it was necessary to adapt the policy as the epidemic progressed rapidly. Shortly after March 16, traffic control bundling was implemented in anticipation of a dramatic increase in pregnant women affected by infection and to better protect the staff. By April 18, with the peak of the COVID-19 epidemic receding, protocols were again readjusted to meet new service delivery requirements. Although a full debrief is yet to occur, from an operational level perspective, staff response was more than satisfactory. While preventing another epidemic may be impossible, this experience will improve our resilience in the future.

During the month of March 2020, health services in France were overwhelmed by the coronavirus disease (COVID-19) pandemic.^1^ To cope with this extraordinary situation, numerous hospitals concentrated health care delivery mainly on resolving the crisis. Consequently, non-urgent consultations and surgical operations were deferred, while ambulatory or home care substituted hospital stays. As this strategy was not applicable to Obstetrics, the COVID-19 infection had to be added to existing clinical care pathways. This was further complicated by an increasing number of infected patients during pregnancy, in addition to the scientific uncertainty about the virus. Moreover, the overall context of fear and anxiety that affected all in society exacerbated challenges,^2^ although lessons to be learned are yet to be fully elucidated because the pandemic is ongoing. This report from the field describes how COVID-19 protocols were adapted to a Parisian maternity unit from an operational managerial perspective.

## ORGANIZATION OF MATERNITY CARE IN NECKER CHILDREN’S HOSPITAL

A specialist referral center located in the center of Paris (*Hôpital Necker–Enfants Malades*), the Necker–Enfants Malades Hospital (children’s hospital) maternity unit is staffed by obstetricians, neonatologists, midwives, nurses, and health care assistants (HCAs).^3^ Equipped with level 3 neonatal intensive care facilities, the department often manages pregnancies complicated by congenital heart defects.

## NECKER CHILDREN’S HOSPITAL COVID-19 INFECTION PREVENTION POLICY

Procedures mitigating infection were devised early on in France during the epidemic by the Necker–Enfants Malades Hospital Infection Prevention and Control team (IPCT).^4^ The first COVID-19 policy written on January 26 detailed isolation and barrier methods for suspected patients (until nasopharyngeal swab polymerase chain reaction [PCR] confirmation). Regularly updated as the situation progressed, this protocol was adapted to maternity care based on scientific recommendations from French and international authorities.^1,5^

Consequently, a separate clinical pathway for a patient with or suspected of having the COVID-19 infection was devised. Upon their arrival at consultations, patients were clinically assessed for symptoms. In the event of a suspected infection, patients were given a surgical mask and their consultation reprogrammed (minimum of 2 weeks’ interval). While telehealth consultations were also proposed, this was not a viable option for patients in the third trimester or with a high-risk pregnancy who required a physical clinical examination. Patients arriving at the maternity birthing suite located on the 5th floor were triaged by HCAs who carried out initial clinical history and temperature measurements. Infected patients (or suspected of the infection) were isolated in an allocated consultation box or birthing room depending on their obstetrical history/clinical symptoms and biological samples taken (blood tests, nasopharyngeal swabs, etc.). Those who required hospitalization for a pregnancy-related pathology were isolated on the maternity ward. For COVID-19 cases that required specialized care, beds were allocated in either the Infectious Diseases Department or adult Intensive Care Unit (ICU).

**FIGURE 1:**
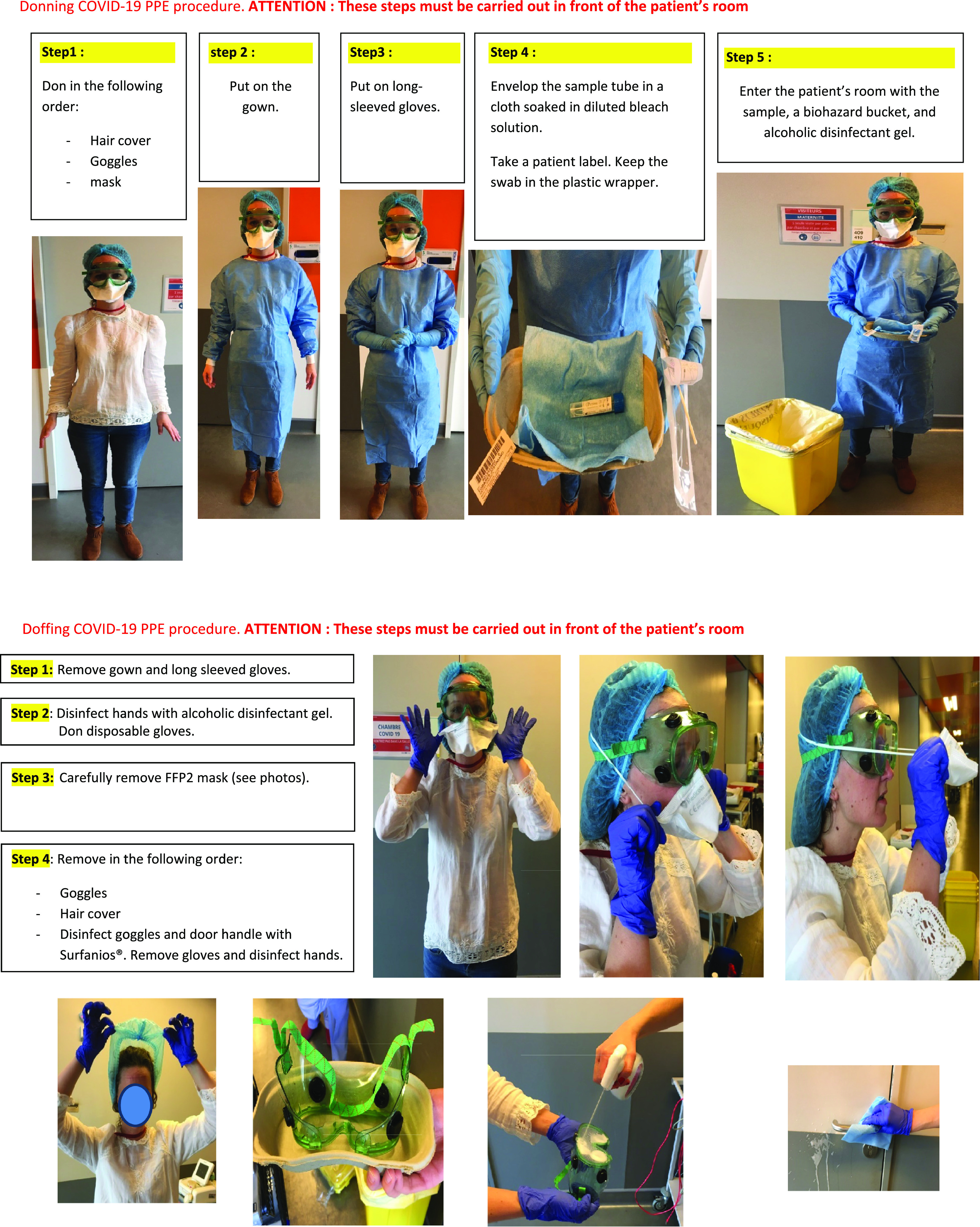
Memo for Donning/Doffing COVID-19 PPE and Disinfection of Biological Samples (Translated from French)

Specific COVID-19 personal protective equipment (PPE) for staff included FFP2 masks, goggles, long-sleeved nitrile gloves, surgical gowns, and hair covers. These were stored on an allocated trolley habitually used for contact isolation precautions (eg, drug-resistant bacterial infections). The trolleys were also equipped with the necessary kit for viral sampling and disinfection. Moreover, staff had to be trained on how to don and doff PPE correctly to avoid contamination (Figure 1). General hospital hygiene of the department was given particular attention, especially the disinfection of door handles every 4 hours.

Furthermore, the policy also outlined procedures for staff suspected of infection, they were screened, and then they were sent home until test results. In the event of confirmed infection, they were included in the COVIDOM cohort and received close medical supervision.

## ADAPTING THE COVID-19 IPCT TO THE EVOLVING CLINICAL SITUATION

Weekly medical manager team meetings involved discussion of the evolving clinical situation (with decisions based on consensus), and an initial evaluation found that our procedure worked well. However, it was necessary to adapt the policy as the epidemic progressed rapidly. This was exacerbated by widespread fear and tension in the days preceding and shortly after the government’s announcement of nationwide confinement on March 16, 2020.^2,6^ As a result of PPE shortages and to prevent black marketing by staff, orders of masks and sanitizing disinfectant gel were centralized by a logistical manager.^2^ Consequently, masks and sanitizing disinfectant gel were ordered and dispensed by managers according to staff needs. This system worked well and enabled to ensure that PPE inventory was adequate throughout the crisis in our hospital. With the announcement of the generalized confinement policy, French hospitals (through the *Plan blanc* hospital emergency contingency plan) canceled leave and rest days, requiring staff to be on-call.^7^ Consequently, there were no staff shortages to deal with this crisis in our department. Midwife managers coordinated staffing requirements, attributing human resources where necessary.

Staff were also affected by the tension caused by widespread fear, anxiety, and confusion that affected the whole population, resulting in the suspension of public transport and childcare facilities. Consequently, to ensure continuous health care delivery, managers organized taxis, hotel room reservations, and childcare according to the roster. A website was also provided to meet the needs of hospital staff (eg, shopping),^8^ while managers provided reassurance and trustworthy scientific/medical information to staff. Psychologists were also present to deal with any psychological trauma triggered by the generalized context of fear, trepidation, and uncertainty caused by the pandemic.

Shortly after March 16, traffic control bundling was implemented in anticipation of a dramatic increase in pregnant women affected by infection and to better protect staff.^9^ All patients with COVID-19 symptoms were tested by 2 dedicated midwives in a diagnostic clinic located in a field tent in front of the hospital entrance. By March 25, a surge in the number of pregnant women infected caused us to rethink clinical pathways again. A specific consultation room manned by an obstetrician attired in PPE to carry out consultations and sonographical examinations of pregnancy was reserved for seropositive patients. Located next to the diagnostic clinic, this also enabled biological samples to be taken by COVID-19 midwives. It was deemed that due to a high proportion of false negatives (30%) and finite resources available, mass diagnostic screening of patients and personnel would not be beneficial in the benefit-harm assessment.^10^ However, at the time of this writing, improved serological, PCR nasopharyngeal rapid diagnostic tests and other diagnostic tests (scanner, ultrasound, etc.) are being developed. Furthermore, a dedicated wing located in an evacuated surgical ward was provided to isolate infected patients and dispense care following delivery. For patients with severe COVID-19 complications, beds were provided in cardiac ICU located on another wing of the 4th floor.

The relocation of COVID-19 patients was aimed to reduce viral exposure to staff and patients on the maternity ward who feared cross exposure. To further limit their risk of contagion, all visits were banned and fathers were confined in the room with their partner and baby until discharge. Furthermore, in line with French obstetrical postnatal care recommendations, patients were discharged early (48 hours after normal delivery and 96 hours after a cesarean section) with a relay by private community midwife care wherever possible.^11^ To prevent COVID-19 complications, anti-inflammatory medication was no longer prescribed systematically for pain relief and substituted for other antalgic drugs (a precautionary measure taken by French authorities based on a few case reports at that time).^12^ Consequently, Midwife managers not only communicated these short-term changes (in team meetings and via e-mail), but also had to be vigilant that protocols were adhered to, adjusting individual behaviors when necessary.

By April 18, with the peak of the COVID-19 epidemic receding, the number of infected patients in France started to decline significantly. It was again decided to readjust protocols to meet new service delivery requirements. Consequently, the allocated isolated COVID-19 wing was closed and the few infected patients were once again hospitalized on the maternity unit located on the 4th floor. The allocated COVID-19 consultations manned by an obstetrician donned in PPE, in addition to the 2 dedicated midwives to carry out PCR diagnostic tests, were also made redundant. In light of new scientific knowledge, anti-inflammatory drugs were reinstated for pain relief.^13^ Once again, staff had to be accompanied with these changes, which were widely accepted and also seen as a shining beacon to the restoration of normality.

## CONCLUSION

Through cohesive teamwork, COVID-19 policies were adapted to maternity health care delivery while mitigating risks to staff and patients. Although a full debrief is yet to occur, from an operational level perspective, staff response was more than satisfactory, illustrated by the fact that only a handful of frontline staff were infected (including 4 managers) in our maternity unit compared to 4180 infected health care professionals in other Parisian hospitals (data for all departments on April 20, 2020). At present, the epidemic is decreasing in France and the rest of Europe; however, the risk of a second wave of infection remains an ever-present foreseeable threat.^14^ To this end, policies are a work in progress and based on French scientific recommendations that are similar to those of other countries faced with the same problems.^1,5,11^ Issues under review include the universal use of masks and testing of all patients who arrive at the maternity unit. This reversal in mindset based on lessons learned in hindsight assumes all to be potentially contagious until proof to the contrary. Furthermore, the serological status of staff is also another point of discussion, the idea being that COVID-19 units be manned by seropositive health care professionals (immunity passports).^15^ The reopening process will also include long-term preventive measures. These will include reducing patient waiting times and ensuring social distancing in waiting rooms and individual rooms on wards. Although preventing another epidemic may be impossible, this experience will improve our resilience in the future.^14^
